# Recruiting hard-to-reach pregnant women at high psychosocial risk: strategies and costs from a randomised controlled trial

**DOI:** 10.1186/s13063-021-05348-9

**Published:** 2021-06-16

**Authors:** Alice MacLachlan, Karen Crawford, Shona Shinwell, Catherine Nixon, Marion Henderson

**Affiliations:** 1 MRC/CSO Social and Public Health Sciences Unit, University of Glasgow, Berkeley Square, 99 Berkeley Street, Glasgow, G3 7HR Scotland; 2 Institute of Health and Wellbeing, Level 4, Academic CAMHS, Yorkhill Hospital, University of Glasgow, Dalnair Street, Glasgow, G3 8SJ Scotland; 3 School of Health Sciences, University of Dundee, 11 Airlie Place, Dundee, DD1 4HJ Scotland; 4 Scottish Children’s Reporter Administration, 10-20 Bell Street, Glasgow, G1 1LG Scotland; 5 Social Work and Social Policy, University of Strathclyde, Lord Hope Building, 141 St James Road, Glasgow, G4 OLT Scotland

**Keywords:** Hard-to-reach population, Marginalised groups, Maternal health, Pregnancy, Randomised controlled trial, Recruitment, Vulnerable groups

## Abstract

**Background:**

Recruiting participants to randomised controlled trials (RCTs) is often challenging, particularly when working with socially disadvantaged populations who are often termed ‘hard-to-reach’ in research. Here we report the recruitment strategies and costs for the Trial for Healthy Relationship Initiatives in the Very Early years (THRIVE), an RCT evaluating two group-based parenting interventions for pregnant women.

**Methods:**

THRIVE aimed to recruit 500 pregnant women with additional health and social care needs in Scotland between 2014 and 2018. Three recruitment strategies were employed: (1) referrals from a health or social care practitioner or voluntary/community organisation (practitioner-led referral), (2) direct engagement with potential participants by research staff (researcher-led recruitment) and (3) self-referral in response to study advertising (self-referral). The number of referrals and recruited participants from each strategy is reported along with the overall cost of recruitment. The impact of recruitment activities and the changes in maternity policy/context on recruitment throughout the study are examined.

**Results:**

THRIVE received 973 referrals: 684 (70%) from practitioners (mainly specialist and general midwives), 273 (28%) from research nurses and 16 (2%) self-referrals. The time spent in antenatal clinics by research nurses each month was positively correlated with the number of referrals received (r = 0.57; p < 0.001). Changes in maternity policies and contexts were reflected in the number of referrals received each month, with both positive and negative impacts throughout the trial. Overall, 50% of referred women were recruited to the trial. Women referred via self-referral, THRIVE research nurses and specialist midwives were most likely to go on to be recruited (81%, 58% and 57%, respectively). Key contributors to recruitment included engaging key groups of referrers, establishing a large flexible workforce to enable recruitment activities to adapt to changes in context throughout the study and identifying the most appropriate setting to engage with potential participants. The overall cost of recruitment was £377 per randomised participant.

**Conclusions:**

Recruitment resulted from a combination of all three strategies. Our reflections on the successes and challenges of these strategies highlight the need for recruitment strategies to be flexible to adapt to complex interventions and real-world challenges. These findings will inform future research in similar hard-to-reach populations.

**Trial registration:**

International Standard Randomised Controlled Trials Number Registry ISRCTN21656568. Retrospectively registered on 28 February 2014

**Supplementary Information:**

The online version contains supplementary material available at 10.1186/s13063-021-05348-9.

## Introduction

Randomised controlled trials (RCTs) are widely accepted as the gold standard for evaluating healthcare interventions and often underpin healthcare decision-making and policies [1, 2]. Recruitment of participants is crucial to the success of RCTs, and one of the greatest challenges for researchers is ensuring that recruitment to a trial is both timely and effective [3–7]. A review of 73 RCTs funded by the UK Health Technology Assessment Programme and the Medical Research Council between 2002 and 2008 concluded that only 55% of trials successfully achieved their original recruitment target and 46% of trials required an extension in order to achieve their funded aim [4].

Failure to recruit enough participants may jeopardise the findings of RCTs, resulting in trials being statistically underpowered or attrition bias causing non-representative sampling. This can lead to false rejection or acceptance of the null hypothesis [8], and should clinically relevant differences between treatment arms be reported as not statistically significant [9], potentially effective treatments may be withheld from patients or delayed while additional evidence is sought regarding effectiveness [6]. In addition, recruiting participants to RCTs is resource-intensive, with a large proportion of the costs of conducting trials associated with recruitment [10]. Trials which fail to reach recruitment targets may seek funded extensions, delaying knowledge transfer to clinical practice and the use of funds that could have been used for other research. Slow recruitment may also adversely affect the delivery of interventions being trialled, particularly if these interventions are delivered using a group format [11]. Therefore, maximising recruitment rates in clinical trials is statistically and financially crucial. Information on the success (or not) and cost of different recruitment strategies can inform the planning of future studies and ensure that more RCTs successfully meet these targets [5, 12]. However, the evidence base for different strategies is currently limited [6, 7, 12, 13].

In recent years, there has been substantial research into improving public health by reducing inequalities in social determinants of health [14]. However, socially disadvantaged groups (e.g. those with low income, lower education levels and a lack of health literacy) are challenging to access, engage and retain in research, and as such are often labelled as ‘hard-to-reach’ populations [15]. RCTs evaluating interventions targeted at hard-to-reach populations therefore face particular recruitment difficulties [15–18], making additional information on successful strategies to recruit such populations particularly valuable.

The Trial for Healthy Relationship Initiatives in the Very Early years (THRIVE) is an RCT evaluating two parenting interventions for pregnant women with additional health and social care needs, each delivered in a group setting [19]. In this descriptive analysis, we present and reflect on the success and estimated cost of the recruitment strategies employed by THRIVE in order to inform future research evaluating complex interventions in similar populations. Given that 46% of recent UK RCTs experienced recruitment challenges resulting in extensions [4], we consider that it is important not only to report on successful recruitment strategies, but also to look at the real-life challenges of recruiting to RCTs and how changes in context within study settings can significantly impact research activities.

## Methods

### Study design and population

THRIVE was a three-arm RCT that evaluated the impact of two parenting interventions on maternal mental health and mother-child interactions in women with additional health and social care needs in pregnancy (trial registration: ISRCTN21656568) [19]. The parenting interventions Enhanced Triple P for Baby and Mellow Bumps were both delivered in addition to care-as-usual and compared with care-as-usual alone.

THRIVE required an 18-month funded extension in order to meet the primary study aims. This was in part due to an 11-month delay in starting recruitment due to contractual issues resulting in belated National Health Service (NHS) research management approval. In addition, after initiating recruitment, one of the interventions changed its delivery format, requiring re-negotiation of costs. It took approximately 10 months to secure new research management approval and funding to deliver the new intervention model. During this time, there was a freeze on research staff recruitment resulting in under-staffing, the impact of which on recruitment is discussed in the results presented here.

The recruitment target was 500 participants. Pregnant women eligible for THRIVE were aged 16 or above (or 14 and above with social work support), living or receiving maternity care within the NHS Greater Glasgow and Clyde and NHS Ayrshire & Arran health boards in Scotland who met one or more of the NHS Greater Glasgow and Clyde Special Needs in Pregnancy (SNiPs) criteria (Table 1) [20]. UK NHS care is universal and free at the point of use, so all potential participants could be accessed through this system and were eligible for the same routine maternity care.

**Table 1:** NHS Greater Glasgow and Clyde Special Needs in Pregnancy criteria

• Alcohol and/or drug misuse in woman and/or partner in the last 12 months • HIV-positive and/or known HIV-positive partner • Current mental health issues • Involvement and/or partner involvement in the criminal justice system • Asylum seeker/refugee • Vulnerable/would benefit from social work support • Current or previously identified child protection issues • Resistant to professional intervention • Learning difficulties that could impact parenting • Domestic violence with child protection issues • Homeless/living in supported accommodation • Vulnerable young mothers, e.g. those accommodated by the local authority or linked to care leaving services, pregnancy under difficult circumstances	

Women were excluded from participation in THRIVE if they were more than 30 weeks pregnant at referral (or reached this point before they could be randomised), lacked capacity to consent to participate in research, had insufficient spoken English to participate in research or engage in groups, had acute mental ill health (e.g. active psychosis), were homeless to the point of being non-contactable, were participating in another trial of an antenatal intervention, if a decision had already been made that their child would be removed at birth or if they miscarried before or after recruitment to the trial. Women who experienced a still birth or infant death were withdrawn from the study.

### Recruitment process

**Fig. 1:**
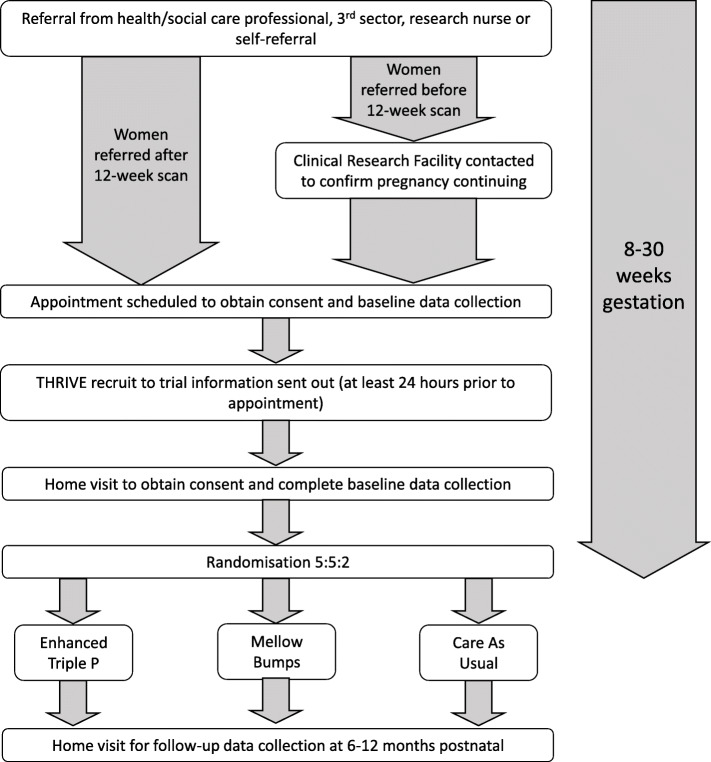
THRIVE recruitment process. Women were recruited between 12 and 30 weeks pregnant but could be referred from 8 weeks

Pregnant women meeting the study eligibility criteria were recruited when they were between 12 and 30 weeks gestation but could be referred from 8 weeks (Fig. 1). Following referral (from a health or social care practitioner, or voluntary/community organisation; research nurse; or self-referral), a member of the THRIVE research team confirmed with the relevant local NHS body whether the pregnancy was continuing. Once confirmed, a member of the THRIVE research team contacted potential participants to arrange an appointment either in the participant’s home or another suitable location, during which the participant would have the opportunity to ask questions about the research and, should they agree, be consented to the trial and complete the baseline assessment. Following completion of the baseline measures, participants were randomised to one of the three trial arms by an independent Clinical Trials Unit, and those randomised to an intervention arm were invited to attend group sessions. Women were given £15 shopping vouchers for the completion of the baseline (and later follow-up) study assessments.

THRIVE employed a team of research nurses to support research staff in the recruitment of participants and consent and data collection procedures. Up to 11 research nurses supported the THRIVE recruitment strategies (see below), by engaging with healthcare professionals at NHS clinics, speaking with pregnant women to gauge eligibility and interest in the study and distributing advertising materials. Research nurses were employed on fractional contracts, in order to provide a flexible workforce and to manage fieldwork demand in a cost-efficient way.

### Recruitment strategies

Pregnant women meeting the study criteria could be referred to the trial in one of three ways.

#### Recruitment strategy 1: Practitioner-led referral (January 2014 to May 2018)

A health or social care practitioner or voluntary/community organisation could make a referral after discussing the trial with the potential participant to gauge eligibility and interest. Referrals could be made in a manner that best suited their working practices: either completing a paper referral form and returning in a prepaid envelope, completing an online referral form, passing on information by email or calling a freephone number. Potential participants were required to provide verbal or written consent for their information to be passed on to the THRIVE research team. Where verbal consent was provided, the referring practitioner had to sign the form to state that consent to share information had been given.

In order to engage health and social care practitioners and voluntary/community organisations and encourage them to refer women to the trial, meetings were held with key stakeholders from relevant organisations at the start of the recruitment period to inform them about the study, the eligibility criteria and recruitment process. Follow-up meetings were held throughout the 4-year recruitment period with both key stakeholders and directly with referring practitioners. These meetings aimed to maintain engagement by providing updates on the changes to processes or eligibility criteria, reminding and encouraging referrers to discuss the study with potential participants and send in referrals, exploring any barriers to recruitment, and identifying other useful contacts. Most engagement meetings were focussed around NHS maternity services, including strategic leads and midwifery teams, although regular meetings were also held with other health and social care professionals and voluntary/community organisations. The trial was supported by key NHS management leads, who assisted in contacting practitioners and disseminating information. In addition to formal engagement meetings, trained members of the THRIVE research team regularly attended maternity clinics to remind practitioners within NHS settings about the study and provide support to identify and engage potential participants. Recruitment materials such as a THRIVE referral flow chart and referral criteria cue cards were developed to support referrers in identifying eligible women, and a study newsletter was regularly circulated within NHS maternity services. Study marketing materials (e.g. pens, highlighters, mug coasters, trolley coin keyrings and canvas bags) with the study logo and contact details for making referrals were given out to potential referrers to act as a reminder of the study. Materials and strategies to support referring practitioners evolved throughout the study to fit in with their working practices based on feedback from interviews with practitioners conducted as part of the study process evaluation.

In addition to the activities above, study information was mailed to all GPs within the study areas (*N* = 407) in early 2014, with subsequent mail-outs of recruitment information and study advertising to a subset of GPs in deprived areas in April 2017 and March 2018. Study information was also emailed to senior obstetricians.

#### Recruitment strategy 2: Researcher-led referral (October 2015 to May 2018)

In October 2015, the study protocol was amended to allow potential participants to be approached by THRIVE research staff (primarily research nurses) directly in clinics or community settings (e.g. GP offices; community groups) to discuss the trial and gauge eligibility. Following consent, a referral form was completed for any women who were eligible and interested in participating in the study, and where possible, the researcher arranged an appointment for the collection of baseline measures.

#### Recruitment strategy 3: Self-referral (January 2014 to May 2018)

Participants could self-refer to the trial. Initially, self-referrals came from participants who had previously spoken to a research team member or referring practitioner about the study but had chosen not to be referred at that point, and then subsequently contacted the study team directly. Advertising materials were approved by the study ethics committee in June 2016 and included posters to be displayed in the community (e.g. community centres, supermarkets, libraries, mother and baby shops) and NHS settings (GP practices, antenatal clinic waiting areas), Gumtree advertisements and Twitter and Facebook posts. Advertisements offered support for women during pregnancy and provided contact details for the research team but did not include details about the study eligibility criteria due to the sensitive and complex nature of the criteria. Eligibility was determined during the initial contact with THRIVE research staff, and if eligible, a referral form was completed.

### Analysis

This is primarily a descriptive analysis of trends in referral and recruitment in response to changes in recruitment strategy and study context over time. Descriptive data are reported for the number of participants referred and randomised to THRIVE and in respect of reasons for ineligibility and non-participation. Quarterly referrals were analysed by recruitment strategy and NHS maternity setting type in order to determine the impact of recruitment activities and changes in maternity policies/contexts, respectively, on referral rate. The conversion rate from referral to randomised participant was analysed by referral source (strategy 1: practitioner-led, including midwives [including specialised midwives for women with additional health and social care needs], other healthcare practitioners, social workers, voluntary/community organisations, other sources; strategy 2: researcher-led; strategy 3: self-referrals). The association between the time spent in clinics and the number of referrals was analysed (Pearson’s correlation coefficient), and participant demographics were compared between referral strategies (analysis of variance for continuous variables; chi-square for categorical variables). Participant demographic data were collected after women provided their consent to participate in the study and are therefore only available for those recruited to the trial and not for all referrals.

The cost of recruitment per randomised participant was calculated based on research staff salary costs for recruitment (based on estimated time spent on recruitment); research nurse salary and travel costs for clinic visits; research nurse salary and travel costs for failed, completed and additional baseline visits; administrative costs for contacting participants to arrange baseline visits, organising staffing rotas for recruitment and baseline data collection activities, organising data collection materials and addressing fieldwork queries related to recruitment; and printing and postage costs for recruitment documentation. Further details of the variables and assumptions for calculating the cost of recruitment are presented in Additional File 1.

## Results

**Fig. 2:**
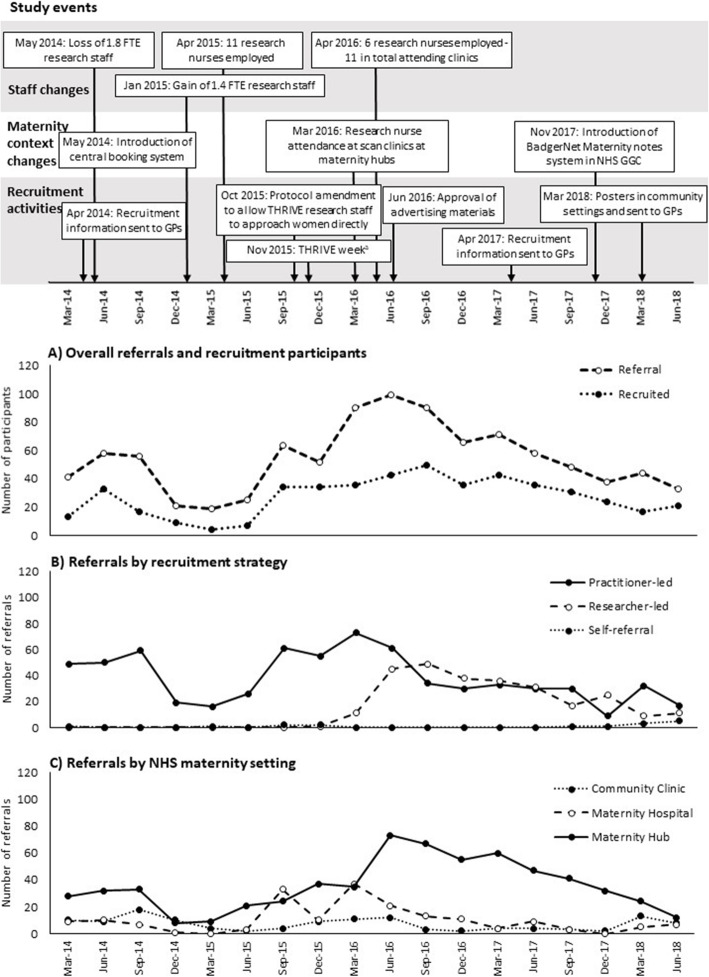
Study events and number of referrals to THRIVE over time. **A** Overall number of referrals and recruited participants. **B** Number of referrals by recruitment strategy. **C** Number of referrals by NHS maternity setting. ^a^‘THRIVE week’ was a week in which THRIVE research staff were present with a stall and recruitment materials at one of the main maternity hubs in Glasgow and in a central Glasgow shopping centre to speak with potential participants and midwives, to determine if a concentrated recruitment drive over a short period would be effective in generating referrals. ^b^Maternity hubs were defined as central hospitals with antenatal clinics, scan clinics and maternity wards. FTE, full-time equivalent; GP, general practitioner

In total, 973 pregnant women with additional health and social care needs were referred to THRIVE (Fig. 2A). The mean number of referrals per month was 18.0 (range 2–44; median 16.5, interquartile range 10–24.5).

### THRIVE recruitment strategies

The effectiveness of the three different recruitment strategies over time is shown in Fig. 2B. In total, 684 practitioner-led referrals (70.3%; strategy 1), 273 researcher-led referrals (28.1%; strategy 2) and 16 self-referrals (1.6%; strategy 3) were received.

#### Recruitment strategy 1: Practitioner-led referral

During the first 24 months of the study, prior to the implementation of recruitment strategies 2 and 3, all referrals came from health and social care practitioners or voluntary/community organisations. Initially, high numbers of referrals were received, following early engagement activities/meetings with midwives, social workers, other health care professionals (primarily health visitors) and 3rd sector organisations. After the initial engagement activities, it became clear that midwives were the main source of referrals for THRIVE among the external organisations initially targeted. As such, subsequent engagement activities, including meetings and clinic attendance, focussed on this group of referrers.

Among the 684 practitioners-led referrals, the majority were received from specialist midwives for women with additional health and social care needs (40.4%) or other midwives (58.0%), with a small percentage received from voluntary/community organisations (0.7%), other health-care professionals (0.4%; health visitors and a clinical psychologist), social work (0.1%) and other sources (0.3%; nursery and early childhood centre) (Table 2). The highest number of referrals per person was from the specialist midwives (27 referrers; mean 10.2 referrals per person).

**Table 2:** Conversion rate from referral to recruited participant by recruitment source

	Referrals (N)	Recruited participants (N)	Conversion rate (%)
***Practitioner-led***			
Specialist midwife^a^ (*n* = 27)	276	156	56.5
Other midwives (*n* = 133)	397	154	38.8
Other healthcare professionals (*n* = 2)	3	2	66.7
Social worker (*n* = 1)	1	0	0.0
3rd sector organisation (*n* = 3)	5	2	40.0
Others (*n* = 2)	2	0	0.0
***Researcher-led*** (*n* = 14)	273	158	57.9
***Self-referral*** (*n* = 16)	16	13	81.3
**Total**	**973**	**485**	**49.8**

*n*, number of people generating referrals; *N*, number of referrals

^a^Specialist midwife for women with additional health and social care needs in pregnancy

Following a reduction in THRIVE research staff in early 2014, there was a subsequent dip in the number of referrals received, as the capacity of the research team to maintain the intensity of engagement activities alongside other study responsibilities was reduced. During this time, recruitment activities ceased in the Ayrshire & Arran region, as this was further from the research base and had a smaller pool of potential referrals. The recruitment of new research staff, and in particular the addition of a team of 11 trained research nurses in 2015 who could attend NHS antenatal clinics on a regular basis, increased the capacity for engagement activities, which was reflected by increasing numbers of referrals from practitioners at this time point (Fig. 2B).

#### Recruitment strategy 2: Researcher-led referral

**Fig. 3:**
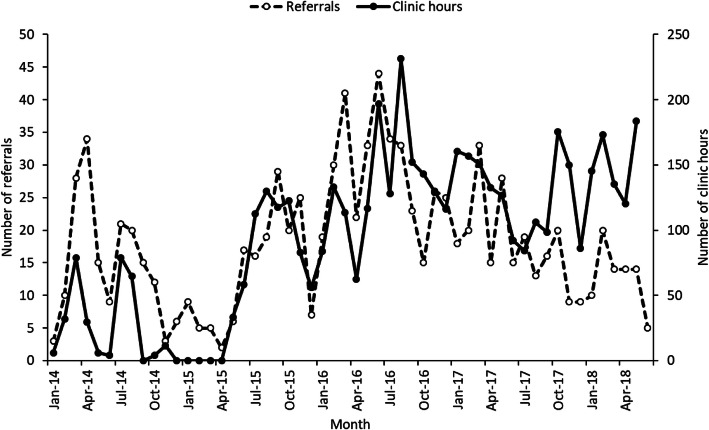
Comparison of monthly THRIVE research nurse clinic attendance and the number of study referrals

The protocol amendment in October 2015 allowing THRIVE research staff (predominantly research nurses) to directly approach women to determine eligibility resulted in a sharp increase in the number of monthly referrals, which then plateaued over time. During recruitment, each research nurse worked between 3.5 and 30 h per week depending on the individual’s availability and demand for fieldwork resource, and overall THRIVE research staff spent an average of 92.9 h per month in NHS maternity clinics. Research staff clinic attendance was positively correlated with the overall number of monthly referrals (r = 0.57; p < 0.001; Fig. 3), suggesting that visibility and engagement with potential referrers and direct engagement with participants were a key driver of recruitment to the study.

#### Recruitment strategy 3: Self-referral

In general, the number of self-referrals was low throughout the study duration. There was a slight increase towards the end of the study, following the display of additional advertising material in community venues, but self-referrals remained lower than the other two recruitment strategies.

### Impact of changes in maternity context on recruitment

Changes in the overall recruitment strategies for THRIVE and the focus of specific activities, such as research nurse clinic attendance and engagement meetings, shifted during the study in response to various factors, including changes in maternity service contexts within the study sites.

The initial recruitment plans were to focus NHS engagement activities on specialist midwife teams who would exclusively see pregnant women who met the THRIVE eligibility criteria. However, in May 2014, NHS sites within Glasgow launched a central booking line system for pregnant women to schedule their initial antenatal appointments, so women who met the SNiPs criteria may not have been directly referred to specialist teams. As a result, engagement activities were diversified early in the study to include midwives and other healthcare professionals based in a wider range of community and hospital antenatal clinics. In addition, discussions with senior midwives revealed that levels of need within the health board area meant that SNiPs midwives only had the capacity to manage those cases with the highest level of clinical need or those where a statutory requirement for additional care was required. Widening recruitment to cover other clinical settings meant that women who met the recruitment criteria but whose cases were not managed by SNiPs teams could be recruited.

The second change to the focus of recruitment activities occurred following a change in maternity policy early in 2016, so that all women attended sonography clinics at ‘maternity hubs’ (maternity hospitals offering central services for antenatal clinics, scan clinics and maternity wards) rather than more local maternity hospitals or community clinics. THRIVE researchers were advised to attend sonography clinics based at the maternity hubs to access potentially eligible women. In addition to providing access to a wider pool of women at one location, targeting women at sonography clinics also had the benefit that by this point, all women had a viable pregnancy (scans conducted at ~ 13 weeks and ~ 20 weeks gestation), which was not always the case when women attended initial booking appointments at antenatal clinics (usually 8–12 weeks gestation). This shift in focus for recruitment resulted in an increase in the number of referrals generated per month, particularly from maternity hubs (Fig. 2C).

The third change within maternity services was the introduction of a new electronic maternity notes system across NHS Greater Glasgow and Clyde in November 2017. The electronic system made it more difficult for midwives to quickly assess patient notes to determine eligibility for THRIVE and limited the ability of sonographers to identify potential participants. As a result, THRIVE research nurses increased their attendance at a wider range of hospital and community maternity clinics, to engage a wider range of midwives rather than focussing on sonography clinics at maternity hubs, and increased efforts at GP clinics and within the community to advertise the study and encourage self-referrals. This shift was reflected in an increase in referrals from maternity hospitals and community clinics towards the end of the recruitment period, although overall the number of monthly referrals decreased (Fig 2C).

### Conversion from referral to recruited participant

Of the 973 pregnant women referred to THRIVE, 485 consented to participate, completed baseline measures and were randomised; an overall conversion rate of 49.8% was generally consistent throughout the study (Fig. 2A). The mean number of participants recruited per month was 9.2 (range 0–19).

Comparison of the conversion rate from referral to recruited participant by recruitment source (Table 2) indicated that although the number of self-referrals was low, 81.3% of these participants were recruited to the study. Conversion rates from specialist midwives (56.5%) and THRIVE research nurses (57.9%) were greater than those for other midwives (38.8%) or voluntary/community organisations (40.0%). No referrals received from social workers and other sources (a nursery and an early childhood centre) resulted in recruited participants, but the number of referrals from these sources was low.

### Characteristics of recruited participants

Participant age, deprivation index (a measure of socioeconomic status), ethnicity, highest educational qualification, employment status, smoking status and number of known additional health and social care needs were significantly different between participants recruited via the three different strategies (all *p* < 0.01; Table 3). Participants recruited from practitioner-led referrals tended to be younger, to live in more deprived areas, have lower educational qualifications, were less likely to be employed and were more likely to be smokers than participants recruited from researcher-led referrals or self-referrals. A lower percentage of self-referred participants was white ethnicity compared with the other groups. While all recruited participants met the study eligibility criteria, those recruited by practitioners had a higher mean number of additional health and social care needs (mean 3.8) versus those recruited by researchers (2.8) or self-referred (2.6).

**Table 3:** Participant demographics based on recruitment strategy (randomised participants only)

Characteristic	Recruitment strategy			***p***-value^**a**^
	Practitioner-led referral, ***N*** = 312	Researcher-led referral, ***N*** = 157	Self-referral, ***N*** = 13	
**Age at randomisation in years, mean (SD)**	25.3 (5.9)	28.6 (6.3)	29.9 (7.4)	< 0.001
**SIMD quintile, n (%)**				
Q1 (most deprived area)	209 (67.0)	82 (52.2)	9 (69.2)	< 0.001
Q2	54 (17.3)	20 (12.7)	0 (0)	
Q3	29 (9.3)	16 (10.2)	0 (0)	
Q4	10 (3.2)	9 (5.7)	1 (7.7)	
Q5 (least deprived area)	10 (3.2)	29 (18.5)	3 (23.1)	
Missing	0 (0)	1 (0.6)	0 (0)	
**Ethnicity, n (%)**				
White	298 (95.5)	145 (92.4)	10 (76.9)	0.001
Asian	4 (1.3)	8 (5.1)	1 (7.7)	
Black	4 (1.3)	3 (1.9)	2 (15.4)	
Others	3 (1.0)	0 (0)	0 (0)	
Missing	3 (1.0)	1 (0.6)	0 (0)	
**Number of pregnancies, n (%)**				
1	142 (45.5)	69 (43.9)	4 (30.8)	0.755
2–3	103 (33.0)	54 (34.4)	5 (38.5)	
4–5	40 (12.8)	25 (15.9)	3 (23.1)	
6+	27 (8.7)	9 (5.7)	1 (7.7)	
Missing	0 (0)	0 (0)	0 (0)	
**Relationship status, n (%)**				
In a relationship	246 (78.8)	135 (86.0)	11 (84.6)	0.458
Separated	10 (3.2)	2 (1.3)	0 (0)	
Single	48 (15.4)	19 (12.1)	2 (15.4)	
Missing	8 (2.6)	1 (0.6)	0 (0)	
**Highest educational qualification, n (%)**				
None	52 (16.7)	16 (10.2)	1 (7.7)	< 0.001
Secondary/vocational	176 (56.4)	54 (34.4)	5 (38.5)	
Higher/A level/HNC/HND	42 (13.5)	30 (19.1)	0 (0)	
Undergraduate degree	12 (3.8)	33 (21.0)	4 (30.8)	
Postgraduate qualification	6 (1.9)	18 (11.5)	3 (23.1)	
Other	5 (1.6)	1 (0.6)	0 (0)	
Missing	19 (6.1)	5 (3.2)	(0)	
**Employment status, n (%)**				
Currently employed	61 (19.6)	86 (54.8)	9 (69.2)	< 0.001
Previously employed	96 (30.8)	20 (12.7)	3 (23.1)	
Never employed	154 (49.4)	50 (31.8)	1 (7.7)	
Missing	1 (0.3)	1 (0.6)	0 (0)	
**Smoking status, n (%)**				
Ex-smoker	102 (32.7)	52 (33.1)	4 (30.8)	0.001
Non-smoker	84 (26.9)	69 (43.9)	7 (53.8)	
Smoker	125 (40.1)	36 (22.9)	2 (15.4)	
Missing	1 (0.3)	0 (0)	0 (0)	
**Number of known additional health and social care needs, mean (SD)**	3.8 (2.0)	2.8 (1.6)	2.6 (1.6)	< 0.001

*Q*, quintile; *SD*, standard deviation; *SIMD*, Scottish Index of Multiple Deprivation

^a^Overall comparison across groups, using analysis of variance for continuous variables and chi-square for categorical variables

### Reasons for non-participation in THRIVE

Among the 485 women who were referred but were not recruited to the trial, 111 did not meet the eligibility criteria, 365 did not consent to participate and 9 were duplicate referrals (Table 4). An additional three women consented to participate but withdrew prior to randomisation. The most common reasons for ineligibility were being more than 30 weeks pregnant at referral (or reached this stage before randomisation; 8.0%) or experiencing a miscarriage or termination of the pregnancy (4.7%). The most common reasons for non-consent were lack of interest in the study (21.0%), being non-contactable (17.7%) or being too busy (13.0%).

**Table 4:** Reasons for non-participation in THRIVE

Reason for non-participation	n (%), ***N*** = 485
***Did not meet eligibility criteria***	***111 (22.9)***
*Exclusion criteria as listed in the study protocol*	
More than 30 weeks pregnant prior to referral to trial^a^	39 (8.0)
Miscarriage or termination	23 (4.7)
Participating in another trial of antenatal interventions	11 (2.3)
Not living in or receiving obstetric/maternity care from NHS GGC or A&A	11 (2.3)
Does not meet 1+ NHS GGC SNiPs criteria	6 (1.2)
Insufficient English to participate in research or engage in groups	6 (1.2)
Lack of capacity to consent to participation in research	4 (0.8)
A decision has already been made that their child will be removed at birth	1 (0.2)
Acute mental ill health	0
Homelessness to the extent of being non-contactable	0
*Additional exclusion criteria*	
Not far enough through pregnancy to complete within the study period	7 (1.4)
Imprisoned after referral	2 (0.4)
Maternal death after referral	1 (0.2)
***Did not consent to participation in the trial***	***365 (75.3)***
Not interested in the study	102 (21.0)
Unable to contact	86 (17.7)
Too busy to participate	63 (13.0)
Failed baseline visits^b^	56 (11.5)
Does not want to participate in research	32 (6.6)
Personal circumstances prevent participation	8 (1.6)
Does not want to attend group sessions	8 (1.6)
Already receiving sufficient support	6 (1.2)
Partner does not support participation	2 (0.4)
Illness/medical reasons prevent participation	1 (0.2)
Feels trial participation is stigmatising	1 (0.2)
***Duplicate referral***	***9 (1.9)***

*A&A*, Ayrshire and Arran; *GGC*, Greater Glasgow and Clyde; *NHS*, National Health Service; *SNiPs*, Special Needs in Pregnancy

^a^Or reached 30 weeks gestation before a baseline appointment and randomisation could be completed

^b^Participant was not at home or did not answer the door when research staff visited for a scheduled appointment

### Resource and cost of recruitment

The overall cost of recruitment for THRIVE was £182,975, equating to £377 per randomised participant (Table 5). The cost of generating the 973 referrals was £128,185, which included 4920 h of clinic time from research staff/nurses across the recruitment period. The cost of converting referrals into randomised participants was £54,790 (including the costs of arranging 195 cancelled/rescheduled appointments, 96 appointments attended by research staff when the participant was not home and 488 successfully completed appointments).

**Table 5:** Resource and costs for recruitment

Cost of recruitment	
*Number of referrals received*	*973*
Total cost of generating referrals	£128,185
Cost per referral	£132
*Number of randomised participants*	*485*
Total cost of recruiting referred participants	£54,790
Total cost of recruitment	£182,975
Cost per randomised participant	£377
**Resource for recruiting referred participants**	
Mean number of contacts required to arrange baseline appointments^a^	2.3
Completed baseline appointments	488
Failed^b^ baseline appointments	96
Cancelled/rescheduled baseline appointments	195
Additional baseline appointment to complete data collection	6

^a^Includes contact with referrals who were not recruited to the study

^b^Participant was not at home or did not answer the door when research staff visited for a scheduled appointment

## Discussion

Over 54 months, 485 pregnant women with additional health and social care needs were recruited to THRIVE, reaching 97% of the target sample size, but requiring an 18-month funded extension. Recruitment to THRIVE was slower than anticipated, with an average of 9.2 participants recruited per month, compared with an original estimate of approximately 20 per month. This was largely due to slow recruitment at the start of the study when the research team had limited resource due to contractual issues, and to changes within the maternity service contexts affecting the recruitment strategies. THRIVE employed three main recruitment strategies, which evolved throughout the study to address recruitment difficulties and respond to the real-life challenges of conducting an RCT of two complex interventions with a hard-to-reach population. Although precise details of the recruitment methods may be specific to the THRIVE study population, the key lessons and overall strategies summarised in Table 6 and discussed below are applicable to a wide range of RCTs, and particularly those recruiting hard-to-reach populations or recruiting within maternity settings.

**Table 6:** Key recruitment lessons for hard-to-reach populations

• Identify and engage with key referrer groups • Use continuous and active recruitment strategies • Ensure research strategies and resource are flexible to adapt to changing circumstances and context • Use study-specific trained recruitment staff (e.g. research nurses) • Identify the most suitable setting in which to approach potential participants	

### Identify and engage with key referrer groups

Initially, a wide range of practitioners across health and social care were targeted with engagement activities to support recruitment to THRIVE. However, as study recruitment progressed, it was found that specialist midwives for women with additional health and social needs had the highest referral rates per person among referring practitioner groups and that women referred by these specialist midwives were more likely to be recruited to trial than women referred from other sources.

Characteristics of the role of specialist midwives that may have made this a key referrer group for THRIVE include their expert knowledge about the THRIVE study population (they almost exclusively see women with additional health and social needs in pregnancy so would require less time screening potential participants and would see a higher proportion of patients eligible for the study) and the fact they usually have a smaller caseload but see women more regularly than other midwives (so would therefore have more opportunity to discuss involvement in the trial and are likely to be seen as a trusted source of information by potential participants as they are able to build a relationship with their patients over time). It is also possible that specialist midwives would have a greater interest in supporting THRIVE, as the outcomes of the study could have direct implications for the future care of their patients.

Other studies have also shown that identifying and engaging with key referrer groups with expert knowledge about the trial or target population are successful strategies for trial recruitment [21, 22]. The importance of involving practitioners in recruiting typically hard-to-reach populations was demonstrated by the differences in characteristics between those referred by practitioners and other referral sources, with those referred by practitioners tending to have higher levels of social disadvantage and more additional health and social care needs. A systematic review of recruitment difficulties in hard-to-reach populations highlighted that mistrust of research or researchers is a key reason for low response rates [15], and a study of recruitment of pregnant women found that the majority preferred to hear about a research study from their healthcare provider who was already seen as a trusted source of information [23].

### Continuous and active recruitment strategies

Several previous studies have found that active recruitment strategies, involving a high level of engagement with research staff, have been most successful in recruiting participants [23–26]. Active strategies for THRIVE consisted of the presence of research staff in NHS clinics and engagement meetings with stakeholders and referring practitioners.

The visibility of study research nurses in antenatal clinics and their direct support in referring potential participants appeared to be a key factor in driving recruitment, with a positive correlation between numbers of clinic visits and referrals. The continued presence of THRIVE research nurses at clinics reduced the burden of study recruitment on already busy healthcare professionals [27] and helped to develop good working relationships with midwives, engendering their continued support throughout the 4-year recruitment period. Other studies have also highlighted the importance of building relationships and referrer buy-in to the success of research study recruitment [23–25, 28].

### Flexibility of research strategies and resource

Another key lesson from THRIVE is that recruitment strategies for RCTs need to be flexible to reflect the realities and changing contexts of complex studies over time. During the THRIVE study, there were several changes in the organisation of maternity services within the study sites, resulting in the adjustment of the focus and reach of engagement activities, particularly clinic visits. Changes in the research staffing model to include a large team of up to 11 research nurses helped the team adapt to changes in maternity systems/contexts. The benefit of employing a large team on flexible contracts, mostly working part time, was that this gave flexibility to cover a wide range of NHS clinics across both health boards, even when clinics were held on the same day. This vastly increased the clinic coverage for THRIVE, resulting in an increase in referrals. Given the long duration of THRIVE recruitment, there were staff rotations within NHS maternity services, and without regular updates and clinic visits, new staff would not have been aware of the study.

### Study-specific trained research nurses

Another benefit of recruiting study-specific staff to support recruitment was that the research nurses were well trained and had both expert knowledge about the study and target population and time to discuss the study in detail with potential participants. This aided research nurses in generating referrals and resulted in a high conversion rate of referral to recruited participant. Spending time with participants and being able to discuss the study in detail also helped build trust between the research nurses and potential participants, which was cultivated throughout the study by continuity between recruitment, baseline and follow-up appointments, where possible. Other studies using a similar approach, with study-specific research staff to support recruitment have also demonstrated the benefits of this [24, 29].

### Identifying the most suitable setting in which to approach potential participants

Identifying central sonography clinics as a suitable setting in which to approach potential participants, and research staff being granted access to these clinics, resulted in a marked increase in THRIVE referral rates. At this point, women would have confirmation that their pregnancy was continuing, and it also provided an opportunity for research nurses to speak to women at a time when they were not receiving other important information from their midwives about their pregnancy. This may have allowed them more time to fully consider whether they wanted to participate in THRIVE when the study was raised at this point. This is supported by information from interviews with referring practitioners from THRIVE [27] and by qualitative data from another UK trial recruiting pregnant women [30]. Sonography clinics also tended to be quieter and provided a better atmosphere for speaking to women about the study.

### Reasons for low referral rates

Although the strategies employed by THRIVE described above did increase the rate of referral to the study over time, the original estimate of recruiting 20 participants per month (based on pilot study recruitment rate [31]) was not reached. At the start of the study, this could partly be explained by resource issues, but may also be due to an overestimate in the number of eligible women who would be referred to the study, and/or an overestimate in the number of referred women who would consent to participate in the study.

An overestimation of the number of eligible participants who will be referred to a study is a common occurrence across RCTs [3]. Based on other literature, potential reasons for under-referral may include healthcare professionals not being aware of the study or not being confident in speaking to potential participants about the study, healthcare professionals acting as gatekeepers to decide whether or not to refer eligible women, lack of time to discuss the study with potential participants and competing interests with other studies in similar populations running concurrently [30, 32–34].

Low recruitment rates within THRIVE are unlikely to be due to a low referral conversion rate, as the reported conversion rate in THRIVE (approximately 50%) was generally similar [35] or higher [11, 36] than other studies in similar hard-to-reach populations within maternity settings, and reasons for non-participation were similar. The provision of £15 shopping vouchers for completion of study assessments may be one reason for the relatively successful conversion to recruited participants, as previous studies have shown that providing a small, but guaranteed, monetary compensation for time is most successful in boosting recruitment to trials, and also aids retention in longitudinal studies [37].

### Cost of recruitment

The total cost of recruitment for THRIVE was £182,975, reflecting approximately 13% of the total study budget, and equating to £377 to recruit each randomised participant. These costs are within the range reported by other studies that employed similar recruitment strategies with a combination of practitioner-led, researcher-led and self-referrals [26, 29].

Some other studies have found passive methods of recruitment more cost-effective, with mail-outs, posters and newspaper advertisements generating high referral rates at a lower cost than researcher- or practitioner-led approaches [38–41]. However, these studies were not recruiting hard-to-reach populations, and for the THRIVE study population, passive approaches (i.e. advertisements for self-referral) were less successful than active approaches (i.e. approach by a practitioner or research staff), highlighting the need for recruitment strategies to consider the study population as well as the cost.

In addition to the cost of recruitment, the persistence needed to contact and arrange appointments with participants from hard-to-reach populations should not be underestimated and needs to be factored in when aiming to recruit a population that is less likely to engage with research and who may have complex lives with many other competing priorities.

### Limitations

A key limitation is that this analysis was based on the referrer name provided on each referral form, but there is likely to have been an overlap between the strategies. For example, some women may have discussed the study initially with a midwife, but then completed the referral procedure with a member of the research staff. This meant that in some cases, it was difficult to distinguish referrals from the three recruitment strategies in order to accurately quantify the number of referrals generated and the cost of individual strategies.

Limitations to the cost analysis include that research staff’s time spent on recruitment and contacting participants for baseline assessment appointments was estimated and may be subject to recall bias, and only research-specific costs were included in the analysis (the NHS support costs of practitioner time to speak to potential participants about the study were not determined). Therefore, the values presented here are an underestimate of the total costs of participant recruitment.

A limitation of the recruitment process for THRIVE was that among referred women who expressed disinterest in participating, the exact reason for the lack of interest was not probed. This information would have been useful to provide a better picture of why some members of socially disadvantaged groups are harder to reach in research.

## Conclusion

Three different recruitment strategies were employed during the THRIVE study: practitioner-led, researcher-led and self-referral. Major factors that contributed to recruitment included identifying and working closely with key groups of referrers, establishing a large flexible workforce to enable recruitment activities to adapt to changes in maternity settings/policies throughout the study period and identifying the most appropriate time and setting at which to discuss recruitment to the study. There was a clear correlation throughout the study between the research staff time/resource available for recruitment activities and the number of referrals received each month.

While the strategies used in THRIVE are largely generalisable to other hard-to-reach populations, by targeting women during pregnancy, THRIVE was able to focus recruitment strategies around an NHS maternity context and target women at a time in their life course when intervention may be more welcome. At other times, and in non-NHS settings, social worker, community group and charity engagement would be important in order to recruit a similar population, and while the overarching strategies described here will be relevant, the method of implementing these strategies is likely to need to be adapted to different contexts.

The recruitment challenges reported in the THRIVE study are not uncommon, particularly when working with hard-to-reach populations and on studies with a long duration during which the study setting and context may evolve. They reflect the need for recruitment strategies to be able to anticipate and adapt to real-world challenges.

## Supplementary Information

**Additional file 1:.** Variables and assumptions for calculating the cost of recruitment to THRIVE.

## Data Availability

The datasets analysed during the current study are available from the corresponding author on reasonable request.

## Abbreviations

A&A: Ayrshire and Arran
GGC: Greater Glasgow and Clyde
GP: General practitioner
NHS: National Health Service
RCT: Randomised controlled trial
SNiPs: Special Needs in Pregnancy
THRIVE: Trial for Healthy Relationship Initiatives for the Very Early years
