# Supplementary material for: Recruiting hard-to-reach pregnant women at high psychosocial risk: strategies and costs from a randomised controlled trial
Source: Trials. 2021 Jun 16;22:402. doi: 10.1186/s13063-021-05348-9 (PMC8207826; doi:10.1186/s13063-021-05348-9)
Supplement: Supplementary file 1 — Additional file 1:. Variables and assumptions for calculating the cost of recruitment to THRIVE. [file 13063_2021_5348_MOESM1_ESM.pdf]

Additonal File 1. Variables and assumptions for calculating the cost of recruitment to THRIVE

Variable	Assumptions	Cost
**Generating referrals**		
Research staff salary costs for time spent on recruitment	Research staff time spent on recruitment included attending clinics, preparation and attendance at engagement meetings with stakeholders and organisation of recruitment activities including research nurse training and rotas and addressing fieldwork queries related to recruitment.<br><br>Salary costs were based on University of Glasgow salary grades for each year of the study, and were calculated based on the FTE of individual staff working on THRIVE and the estimated percentage of time each member of staff spent on recruitment for each month of the study. Due to changes in staffing levels throughout the study, and the other roles required by research staff (e.g. developing materials, data collection, analysis), the overall amount of research staff time available to support recruitment varied from 0.3–1.0 FTE throughout the study.	£64,300
Research nurse salary costs for clinic visits	The total number of hours spent in clinics by research nurse was calculated and costs were based on the average research nurse salary.	£55,860

Research nurse travel costs for clinic visits	Mileage was not paid for clinic visits, but parking costs at hospitals could be reimbursed. Parking costs were calculated based on expenses claims.	£2,730
Recruitment materials	Printing costs for a range of recruitment materials including: leaflets, posters, flyers, information sheets for health-card professionals, referral forms, referral criteria, pre-paid envelopes to return referrals forms, appointment cards, newsletters and marketing materials (pens, bags, coasters).	£5,295
**Recruiting referred participants**		
Research staff/research nurse salary costs for completed baseline assessments	In total, 488 baseline assessments were completed. Baseline assessments were conducted as home visits to participants, and 6 participants required an additional visit to complete all of the baseline assessment. Due to changes in staffing throughout the study, home visits were conducted by research staff at earlier time points in the study and by research nurses later in the study – the proportion of visits conducted by group was determined based on study records. Salary costs for completed baseline assessments were based on the average number of hours for each home visit plus travel time and the average hourly rate for research nurses and Grade 6 research staff. Based on records from 2018, approximately 50% of visits were attended by two members of staff and 50% by one member of staff.	£35,249

Research staff/research nurse salary costs for failed baseline assessments	In total, 118 scheduled baseline assessment appointments did not go ahead but incurred costs due to research staff attending the participants home. In these cases the participant was not at home or did not answer the door, but had not cancelled or rescheduled the assessment in advance. As above, home visits may have been attended by research staff or research nurses, and the costs were based on the average number of hours and travel time and average salary cost, with 50% included as two-person visits.	£3,938
Travel expenses for baseline assessments	Travel expenses were based on the mileage to each home visit for completed and failed baseline assessments and the University of Glasgow mileage expenses claim policy.	£6,361
Administrator salary costs for arranging appointments and organising baseline assessment materials	Administrator staff time spent on recruitment included contacting participants to arrange baseline assessment appointment and organising baseline assessment materials. The amount of time spent on these tasks was calculated based on the total number of contact attempts at baseline to both recruited and non-recruited participants (estimated at 5 minutes per contact attempt), and the number of assessment material packs required for planned baseline appointments (esimated at 10 minutes per pack). Salary costs were based on a Grade 4 staff for the University of Glasgow for each year of the study.	£4,370

Baseline assessment materials	Printing costs for participant information, consent and baseline assessment materials included: participant information sheet, consent form, contact details form, baseline questionnaire (56 pages), service use diary and a useful contacts leaflet.	£4,872
